# Emergent Stem Cell Homeostasis in the *C. elegans* Germline Is Revealed by Hybrid Modeling

**DOI:** 10.1016/j.bpj.2015.06.007

**Published:** 2015-07-21

**Authors:** Benjamin A. Hall, Nir Piterman, Alex Hajnal, Jasmin Fisher

**Affiliations:** 1 Medical Research Council Cancer Unit, Hutchison/Medical Research Council Research Centre, University of Cambridge, Cambridge, United Kingdom; 2 Microsoft Research Cambridge, Cambridge, UK; 3 Department of Computer Science, University of Leicester, Leicester, UK; 4 Institute of Molecular Life Sciences, University of Zurich, Zurich, Switzerland; 5 Department of Biochemistry, University of Cambridge, Cambridge, UK

## Abstract

The establishment of homeostasis among cell growth, differentiation, and apoptosis is of key importance for organogenesis. Stem cells respond to temporally and spatially regulated signals by switching from mitotic proliferation to asymmetric cell division and differentiation. Executable computer models of signaling pathways can accurately reproduce a wide range of biological phenomena by reducing detailed chemical kinetics to a discrete, finite form. Moreover, coordinated cell movements and physical cell-cell interactions are required for the formation of three-dimensional structures that are the building blocks of organs. To capture all these aspects, we have developed a hybrid executable/physical model describing stem cell proliferation, differentiation, and homeostasis in the *Caenorhabditis elegans* germline. Using this hybrid model, we are able to track cell lineages and dynamic cell movements during germ cell differentiation. We further show how apoptosis regulates germ cell homeostasis in the gonad, and propose a role for intercellular pressure in developmental control. Finally, we use the model to demonstrate how an executable model can be developed from the hybrid system, identifying a mechanism that ensures invariance in fate patterns in the presence of instability.

## Introduction

Organogenesis in multicellular organisms is a highly reliable process, achieved by robust temporal and spatial signals transmitted and received by cells within a tissue. In this process, populations of mitotic and apoptotic cells within an organ achieve homeostasis. The movement of cells in a growing organ, triggered by cell division or death, may initiate signaling events and differentiation—thereby coupling controls explicitly to the cellular dynamics.

**Figure 1:**
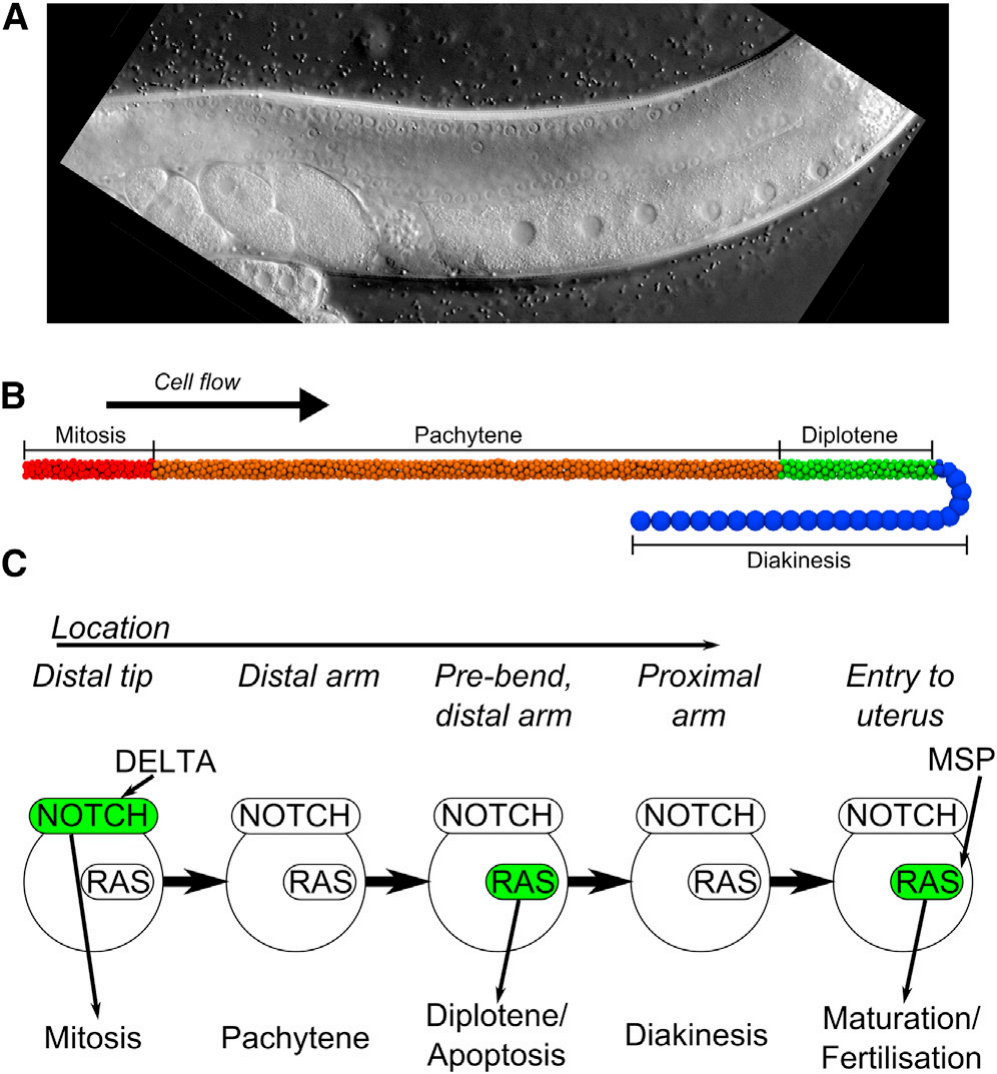
Images of the *C. elegans* germline and our model. (*A*) Microscopy images of the germline. (*B*) The complete physical model of the germ cells. Cells in the distal tip undergo mitosis in response to DELTA, and movement out of the distal tip initiates cell behavior shifting to meiosis and entry into pachytene stage I (*orange*). RAS activation in the pachytene region causes progression through pachytene and entry into diplotene (*green*). In the bend region, germ cells progress into diakinesis (*blue*), begin oogenesis, and move into the proximal arm. The first 150 oocytes are fertilized at the end of the proximal gonad arm and removed from the simulation. Instead of progressing through diplotene/diakinesis, approximately half of the germ cells undergo apoptosis. After fertilization has ceased, the tube becomes blocked and oocytes cease moving. (*C*) A simplified model of fate progression in germline cells. DELTA activates NOTCH, triggering mitosis. Once a cell moves out of a DELTA-rich environment, cells enter pachytene where RAS activation causes pachytene exit and entry into diplotene or apoptosis. As RAS is downregulated, cells progress into diakinesis. Finally, the major sperm protein (*MSP*) induces oocyte maturation in the most proximal oocyte by reactivating the RAS/MAPK pathway.

An organ exemplifying this problem of multiscale control of development is the *Caenorhabditis elegans* germline (Fig. 1 *A*) (1–3). The *C. elegans* gonad is formed by a pair of U-shaped tubes that are each connected with their proximal ends to a common uterus. In the distal region of each gonad arm, germ cells form a multinucleate syncytium, in which the germ-cell nuclei line the outer gonad perimeter and each nucleus is partially enclosed by a plasma membrane but connected by a shared cytoplasm (i.e., the rachis) that fills the inner part of the distal arm. In the bend region, which connects the distal and proximal gonad arms, the germ cells become cellularized and start oogenesis. As the differentiating, immature oocytes enter the proximal arm, they then grow in size, become stacked in single-file, and proceed toward the uterus. This process is controlled by the local signaling molecules present in different regions of the gonad. At the distal tip of each arm, a DELTA signal from the somatic distal tip cell activates NOTCH signaling to promote mitosis and establish a pool of regenerating stem cells (4–7). As this stem cell niche fills, mitotic cells move out of the distal zone and no longer receive the DELTA signal from the distal tip cell. As a consequence, the cells enter meiosis (8,9). Continued pressure from mitotic division in the distal zone drives meiotic germ cells toward the bend region at the end of the distal arm. RAS/MAPK signaling is activated in the distal arm to promote progression through the pachytene stage and entry into diplotene (10–14). Finally, as the cells move through the bend into the proximal arm they enter diakinesis, turn off RAS/MAPK signaling, cellularize, and grow in size to form oocytes. However, it has been estimated that at least 50% of all germ cells undergo apoptosis at the end of the distal arm near the bend region, instead of initiating oogenesis (15,16). Hyperactivation of the RAS/MAPK signaling pathway causes—directly or indirectly—an increased rate of apoptosis (17–19). The immature oocytes in the proximal arm move toward the spermatheca at the proximal end, where a sperm signal induces oocyte maturation and cell cycle progression by reactivating the RAS/MAPK pathway. Thus, germ cell homeostasis is achieved by the competition of mitosis, fertilization, and apoptosis, which maintain a steady number of germ cells. This progression of states, mitosis → pachytene → diplotene → diakinesis, from the distal tip region up to the proximal gonad end, is invariant in the wild-type (20). Uniquely in *C. elegans*, mutations that activate germ cell mitosis lead to the formation of germ-cell tumors (20).

The development of cells in *C. elegans* germline is therefore controlled by the intersection of both physical forces exerted between cells and the internal signal transduction networks acting within individual cells. Models of the germline must therefore capture both of these phenomena to accurately describe the process. Executable models (also known as formal models) have been established as a powerful technique for describing cellular signaling networks (21–24). In contrast to other types of models that aim to represent a literal representation of physico-chemical properties, executable models capture the underlying function of the cell in a more abstract description. In modeling the functional behavior of proteins and genes in a cell (20), we derive a finite, discrete model of development, which accurately describes observed behaviors (25–27). Such models have the further advantage of being amenable to model-checking approaches. These methods offer guarantees of model behavior through analytical techniques, while avoiding the need for explicit exhaustive simulation (28,29). Despite their successes, however, executable approaches cannot be easily applied to three-dimensional biophysical systems. Previously, Beyer et al. (30) showed that a molecular dynamics approach could be used in a physical model of the *C. elegans* germline to describe the physical motions of growing cells. In our approach, we applied the principles of Brownian dynamics to model the movement of entire cells in time and space. Brownian dynamics is a lattice-free, physically realistic implicit solvent model of physical motion originally developed for atomically detailed systems. Particles in Brownian dynamics simulations have no momentum due to a high friction environment, and as such offer a powerful framework for considering the motions of large entities such as cells. Moreover, it is being increasingly applied to the analysis of micrometer-scale cellular and physical systems (such as polymer/clay nanocomposites (31), and rheological systems (32), cell swimming (33), and cell adhesion (34)), demonstrating its appropriateness for considering the dynamics of developing cells in a tissue. To accurately describe the development of the *C. elegans* germ cells, we have combined these two types of model (physical and executable) into a single hybrid, multiscale model. To link the physical and executable models, one requires an interface between the two models describing how spatial location and biophysical properties of the cells influence their signaling states, and vice versa. This interface defines how cells grow, divide, die, and respond to external signals.

Here we present such a hybrid approach for modeling the development of oocytes from stem cells in the *C. elegans* gonad. We take two standard methods to describe executable signaling behavior (using qualitative networks (35)) and physical movements (using Brownian dynamics (36)), and combine them into a hybrid model. Using this hybrid model, we investigate the mechanisms preventing clonal dominance, the role of apoptosis in maintaining germ cell homeostasis, and the control of fate progression through directional flow.

## Materials and Methods

### Cells in a hybrid model

**Figure 2:**
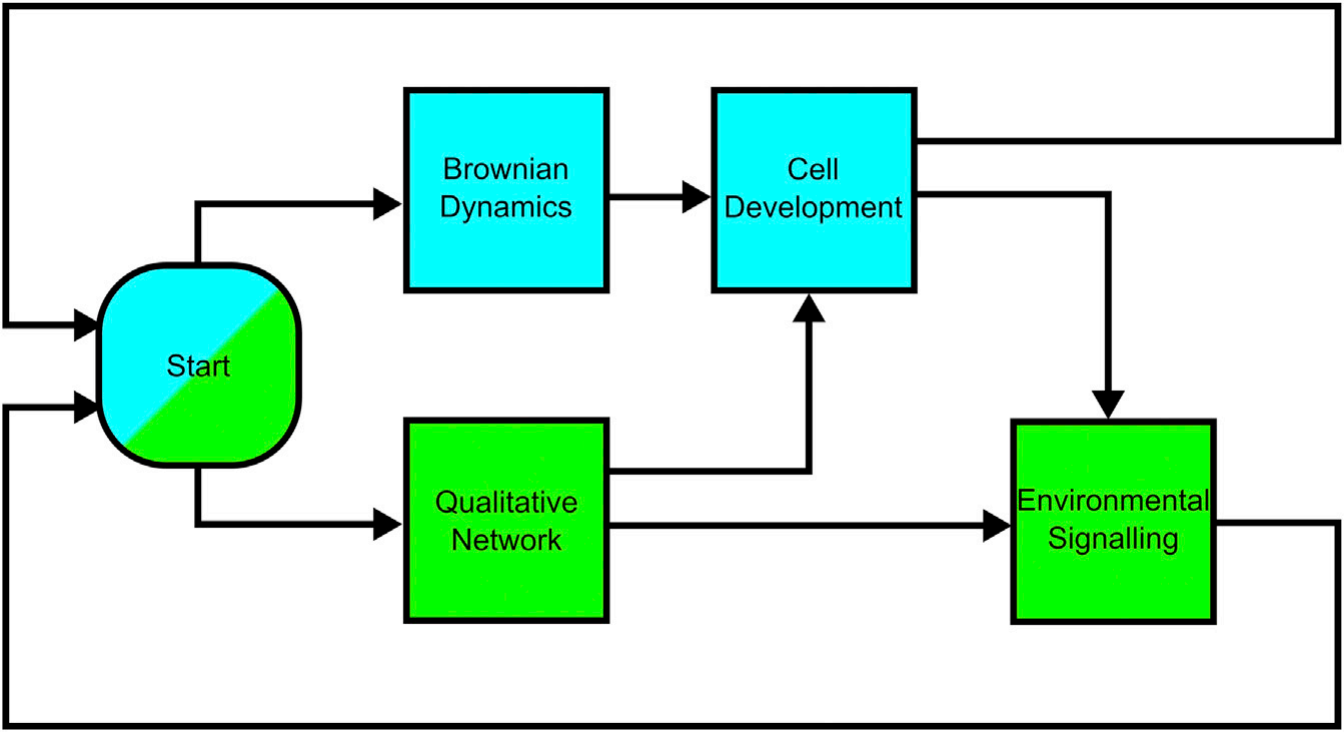
Depiction of the hybrid model updates as a flow chart. The hybrid model is made of a set of cells, each of which consists of a physical particle and a single state of a QN. (*Blue*) Updates to the physical models; (*green*) updates to the QN. Initially, both the cell positions and QNs are updated independently, based on their previous state (see the main text for Brownian Dynamics, and Qualitative Networks). The cell particles are then updated based on the cell’s physical properties and the QN (cell development), to account for growth, division, and death of the cells. Cells that divide are replaced by two cells whose total volume is equal to the volume of the parent cell, and each with a QN in the same state as the parent. Finally, the QN is updated according to the new positions and physical properties of the cell. To see this figure in color, go online.

Each cell in the system consists of a single particle and a single qualitative network (QN) (35). Particles describe the physical parameters, including location in space, whereas the QN describes the signaling state of the cell. At each time step of the simulation, the state of the system is updated. A system update consists of all, some, or none of the following functions applied to the system: an update of the QN (following its formalism defined below); an update of the particle (using a Brownian dynamics integrator); and a hybrid update (changing the QN according to some state of the particle and vice versa, see Fig. 2). Each of these updates is described below.

### Qualitative networks

A quantitative network (QN) is formally defined as follows: a QN, *Q* = (*V*, *T*, *N*), is described by a set of *m* variables *V* = [*v*_1_, …, *v*_*m*_], which can each have integer values in the range [0, …, *N*] and are each associated with a single target function *T* = [*T*_1_, …, *T*_*m*_]. The value of each variable corresponds to the quantity of a substance in the cell, or the activity of a substance in the cell (i.e., 0 = off, *N* = maximum activity), and this granularity can differ between variables. For example, a variable representing VAB1 can have a value of 0 or 1, representing the off-/on-states of the protein. The target function associated with each variable describes how the quantity or activity of the substance varies gradually based on its activators and inhibitors. For example, in the model the function of NOTCH is dependent on the value of the DELTA variable: if DELTA is equal to 1, the value of Notch in the next step is equal to 1. The default target function is calculated from the difference between the average of the activating inputs and the average of the inhibiting inputs (i.e., the average difference). A state of the network *s* is a value for all *V* in *Q*, and all states are considered initial states.

The update function for a QN is defined as follows from state *s* = (*d*_1_, *d*_2_, …, *d*_*m*_). Here, each *d* is the value of a variable at a point in time, i.e., a state of the system is an assignment of a single value to each variable. The next state *s*′ = (*d*_1_′, *d*_2_′, …, *d*_*m*_*′*) is computed by updating each variable as

$$\begin{array}{l} d_{\upsilon }^{\prime }=d_{\upsilon }-1\;\text{if}\;T\upsilon \left(s\right)<d_{\upsilon }\;\text{and}\;d_{\upsilon }>0,\\ d_{\upsilon }^{\prime }=d_{\upsilon }+1\;\text{if}\;T\upsilon \left(s\right)>d_{\upsilon }\;\text{and}\;d_{\upsilon }<N,\\ d_{\upsilon }^{\prime }=d_{\upsilon }\;\text{otherwise}. \end{array}$$

That is to say, for a specific variable, the next state of that variable is either the same, one higher, or one lower. If the evaluated target function (which takes the value of specific variables from the state of the system *s*, hence *Tυ*(*s*)) is higher than this value, and this value is lower than the upper bound, the next state of the variable is this state increased by 1. If the evaluated target function is lower than this state and this state is higher than the lower bound, the next state of the variable is this state is reduced by 1. In all other circumstances, the next state of the variable is equal to the previous state.

All variables update concurrently. Thus, all executions end in a cycle of states that are visited infinitely often. If all executions end in a single cycle, and that cycle is of length 1 (i.e., *T*(*s*) = *s*, therefore *s*′ = *s*), we conclude that the network is stabilizing. The final state in a stabilizing network is known as the stable state.

The QN model was developed in the BioModelAnalyzer (BMA) (37).

### Brownian dynamics

Physical simulations were performed using a Brownian dynamics simulation (36). Cells are considered to have no long-range interactions. Short-range interactions are modeled as harmonic repulsion when cell boundaries overlap (with spring constant 36 pN/*μ*m, estimated based on experimental data from fibroblasts (38)). Germ cell positions are updated according to the Brownian dynamics equation

$$x^{\prime }=x+dt.F.\left(\gamma /m\right)+\text{sqrt}\left(2.k_{\text{B}}T.dt.\left(\gamma /m\right)\right)r,$$

where *dt* is the time step, *F* is the sum of the forces experienced by a cell, *γ* is the friction coefficient, *m* is the mass, *k*_B_ is the Boltzmann constant, *T* is the temperature, and *r* is Gaussian-distributed noise with a mean of 0 and a standard deviation of 1 (taken from the GROMACS manual (39,40)). Temperature was set at 298 K, and the friction coefficient (*γ*) was set to 5 fs (estimated from simulation). Based on the density of an *Escherichia coli* cell, particles were estimated to have a density of 1.3 pg/*μ*m^3^, and cell mass (*m*) was calculated from the cell radius and density. A variable timestep was used with a maximum of 3 s.

### Hybrid updates

Every 0.3 s, both the physical model and the QN for each cell are updated. QNs are updated based on the location of the associated particle in space, or the amount of (physical) time that a variable in the QN has been in a certain state. If the particle is in one of a set of defined regions, the value of a single variable is changed. In the germline model, three regions are defined as DELTA, Growth factor, and Sperm, which represent the Notch activating region, the RAS activating region, and the fertilization region. An alternative model exchanged the RAS activating region with a timed activation and downregulation of RAS (represented by a transition of the Boolean variable from 1 to 0) based on time spent in the meiotic and differentiating states.

Physical particles are updated based on both the state of specific variables in the QN and the physical environment. Cells can grow, shrink, die, or divide. Cells that divide replace the parent cell with two new particles and QNs. Daughter QNs have the same state as the parent QN, and the new particles have the same volume as the parent particle and are both displaced along a defined orientation vector (a property of the parent particle), which crosses the center of mass of the parent particle. Cell growth is modeled as a linear increase in cell radius over time. Cells that both grow and divide grow linearly until they reach a defined maximum size threshold, after which they are replaced by two new cells whose sum volume is equal to that of the parent. The axis of division is initially assigned to cells arbitrarily, and changes direction over time due to Brownian motion. Variability in cell division length is modeled by randomly modifying the size threshold for individual cells according to a normal distribution with known properties. Cell shrinking occurs as a linear reduction in cell radius over time, and cell death occurs once cells reach a defined minimum size. The probabilities of random events (e.g., cell shrinking and cell death, see below) are calculated for a given timestep from a user-defined period of time, and the user-defined probability of that event occurring in that time period.

### Visualization

Cell positions and sizes were visualized using the software VMD (41) for analysis. States of individual cells were visualized with simulation trajectories in VMD using TCL scripts. Logs of births and deaths were converted into Newick format and visualized as phylogenies using the programming language R (http://www.r-project.org/). Plots of rates of mitosis, apoptosis, and fertilization show that the model predicts fertilization rates within an order of magnitude of the experimentally observed values (Fig. S1 in the Supporting Material).

## Results and Discussion

### A hybrid model for germline development in *C. elegans*

In the germline, mitotic cell divisions induced by NOTCH signaling in the distal region generate forces that drive cells away from the distal tip toward the bend region where oocyte differentiation is initiated. These forces initially drive a front of cells out of the distal tip zone, causing the cells to become meiotic as they move along the tube. Activation of RAS/MAPK (10) signaling in late pachytene as cells approach the bend region triggers entry into diplotene (Fig. 1). As cells move into the bend region, MAPK is downregulated and the germ cells enter diakinesis and develop into cellularized oocytes. However, at least 50% of all germ cell undergo apoptosis instead of forming oocytes. As the oocytes reach the proximal end of the gonad arm, a sperm-derived signal induces oocyte maturation by reactivating the RAS/MAPK pathway in the proximal-most oocyte. Finally, the mature oocytes exit the gonad through the spermatheca, where they are fertilized, and enter the uterus.

**Figure 3:**
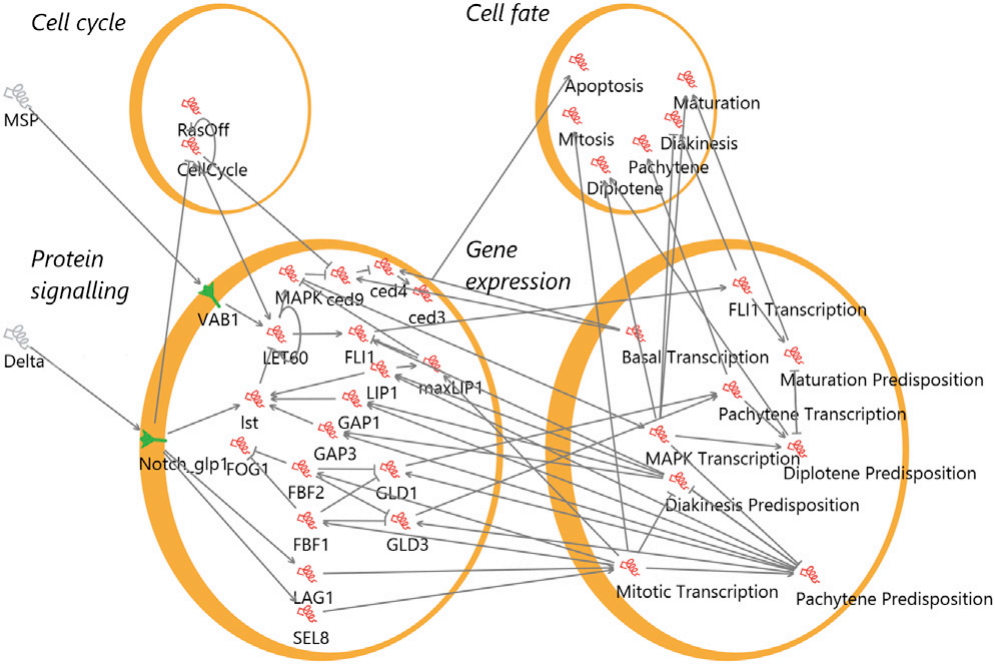
QN model of germ cell signaling, rendered in the BMA. Cell fates, cell cycle events, protein signaling networks, and gene expressions are represented as separate entities for clarity. Each gene, protein, or fate is a separate variable in the QN. To see this figure in color, go online.

To capture the different steps of germ cell development, cells in the hybrid model have two components: a physical particle described using Brownian dynamics, and a signaling state described using qualitative networks (Fig. 3). The dynamics of a cell are controlled by the physical forces exerted on the particle and Brownian motion, arising from the collision of atoms with particles (also known as random thermal motion), which vary depending on the mass of the particle. The particles additionally have an internal orientation, which is updated by thermal motion over the course of the simulation and dictates the axis of division. The walls of the gonad and the syncytium are modeled as static particles, forming a barrier, which ensures cells form a monolayer until the end of the distal arm. The gonad structure itself is capped at the proximal end of the gonad, where the arm would lead to the uterus. The size and shape of this structure was based on experimental microscopy images. Simulations start from a single cell in the distal tip region, and run for 21 days, representing the lifetime of the worm. The signaling state of the initial cell is an arbitrarily selected state, and changes according to the presence of local ligands and the QN formalism. When the gonad is filled with germ cells, it contains the expected number of germ cells (∼1000) (Fig. 1 *B*).

Communication between the physical and signaling models occurs through an interface update. This interface update consists of a physical update, where the state of the QN model changes the property of the physical model, and an executable update, where properties of the physical particle change the executable model. In our model, three signaling regions are defined for the executable update. These are cuboid sections of space, representing external ligands, which alter individual variables in the executable model, if a cell enters them. These three regions represent the presence of DELTA in the distal tip region (the DELTA zone); the presence of a RAS activating ligand near the end of the distal arm, before the bend (the RAS zone); and the presence of sperm/MAPK activating ligands at the end of the proximal arm (the Fertilization zone) (Fig. 1, *B* and *C*). In the physical update, cells may grow, divide, and die. Cells that are in mitosis grow over a period of ∼20 h, until they divide into two smaller cells, which each inherit the parent cell’s signaling state. At least 50% of cells undergo apoptosis, and are therefore removed from the simulation. Two distinct mechanisms of cell death are explored: a single-step model, where cells randomly die and are instantly removed from the simulation; and a multistep model, where cells shrink to a threshold size before death and removal from the simulation. Cells that leave the RAS factor region and have not entered apoptosis grow if the pressure from external forces on the cell is below a defined threshold. Finally, the first 150 oocytes that enter the maturation state are removed from the simulation at the proximal gonad end to represent oocyte fertilization.

### Predictions arising from the model

#### Mixing of mitotic stem cells avoids clonal dominance

**Figure 4:**
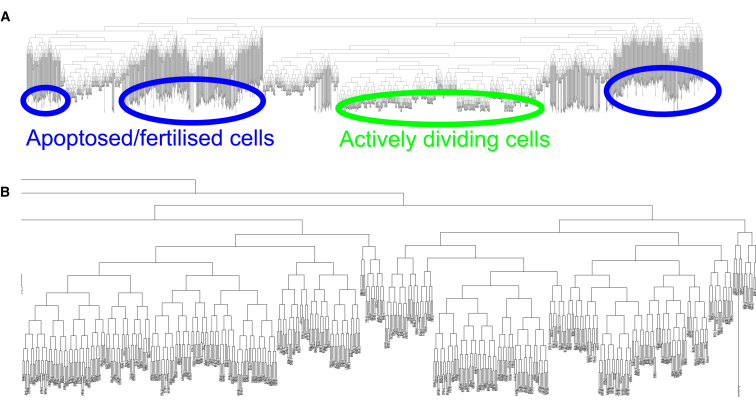
Simulated lineage of germline cells. (*A*) The complete lineage of model germline cells descended from a single cell over 21 days. Cell divisions are indicated by a fork in the lineage. (*Long*, *vertical shaded lines*) Cells have stopped dividing for a period of time and been removed from the simulation (by death or fertilization). Fertilization and apoptosis can be seen to remove entire branches of the lineage, but at any one time the pool of proliferating cells is made up of a number of different branches due to thermal mixing (the randomization of mitotic planes by Brownian motion). (*B*) Expanded lineage showing just the population of dividing cells at the end of the simulation. The final set of dividing cells is separated by up to 13 generations. Seven to eight generations would be sufficient to generate ∼200 cells to fill the distal tip. To see this figure in color, go online.

**Figure 5:**
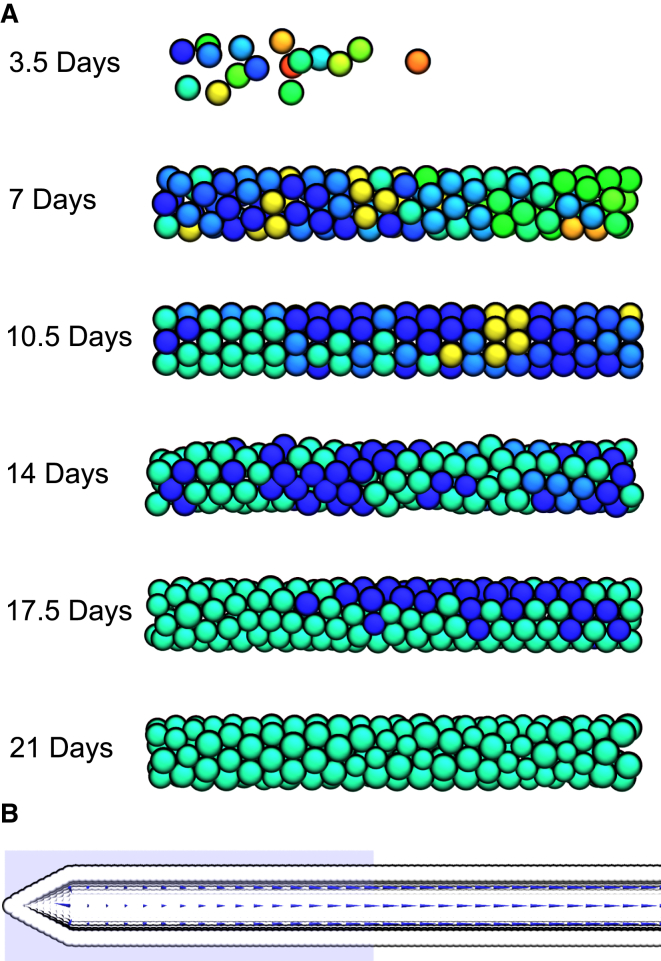
Dynamics of the stem cell population. (*A*) At 3.5 days, every cell is given a unique color and all descendants of that cell retain the same color. In the model, it takes ∼18 days for ancestors of one of these cells to exclusively dominate the distal tip (roughly 22 generations). In principle, the descendants of a single cell could dominate within eight generations (7 days). (*B*) The vector field of average cellular motion across 21 days (plotted as *blue spikes*). (*White*) Cross section of the gonadal wall; (*blue box*) distal tip region. In the mitotic region, the forces generated by cellular division cause cells to move randomly, which in turn causes the averages to be small and/or directionless. Cells in the pachytene stage and at the edge of the distal tip zone move clearly in a single direction, driven by forces generated through division in the distal tip.

Clonal dominance is the process by which single cell lineages come to dominate a population of cells. This plays a role in the development of cancers, where single mutated stem cells reproduce and eventually make up the majority of the population of growing cells, increasing the likelihood of further mutation and ultimately tumor development. In the case of germline stem cells, clonal dominance would be detrimental because it would reduce genetic diversity and propagate harmful mutations. In human systems, the accumulation of harmful mutations that may result from clonal dominance would be expected to increase the likelihood of cancers based on the Vogelstein model of tumor development (42). Given the low likelihood of a cell entering carcinogenesis, it is reasonable to propose that mechanisms exist to prevent this, although such mechanisms are not presently known. Through our model, however, the lineages of the cells can be tracked and plotted, either as a phylogeny (Fig. 4), or visualized in three-dimensional space (Fig. 5 and Movies S1 and S2 in the Supporting Material), allowing us to analyze this behavior directly. Examination of these graphics highlighted several unexpected emergent features. Firstly, whole branches defined after a limited number of divisions can be seen to stop dividing and either die by apoptosis or be fertilized. However, the stem cell population at the end of a 21-day simulation consisted of a handful of separate branches, separated by up to 13 generations. This would suggest that the probability of any single cell coming to dominate the germline is relatively low, but that the pool of stem cells remains relatively diverse (compared to the eight divisions that are sufficient to fill the distal tip).

Closer examination revealed that, while all germ cells in the simulation are undergoing thermal motion, the type of motion varies along the length of the gonad. Mitotic cells undergo greater lateral motion around the wall of the tube, while other cells move almost exclusively along the tube’s length (Fig. 5 *B*). This arises from the randomized orientation of the mitotic cleavage planes due to Brownian motion, combined with the forces generated in mitosis, which allows lateral motion along the vector of the cell orientation. The movements of cells that enter meiosis are driven by the forces along the gonad in a single direction, and so undergo less lateral movement. We propose that this increased lateral motion effectively mixes the stem cell population, and that this mixing in turn acts as a barrier to clonal dominance. A deleterious mutation in a single germ cell, which does not alter division rate, is therefore unlikely to dominate the stem cell niche due to this thermal mixing, making the genome more robust to mutagenesis.

#### Apoptosis reduces cellular flow by killing small cells

**Figure 6:**
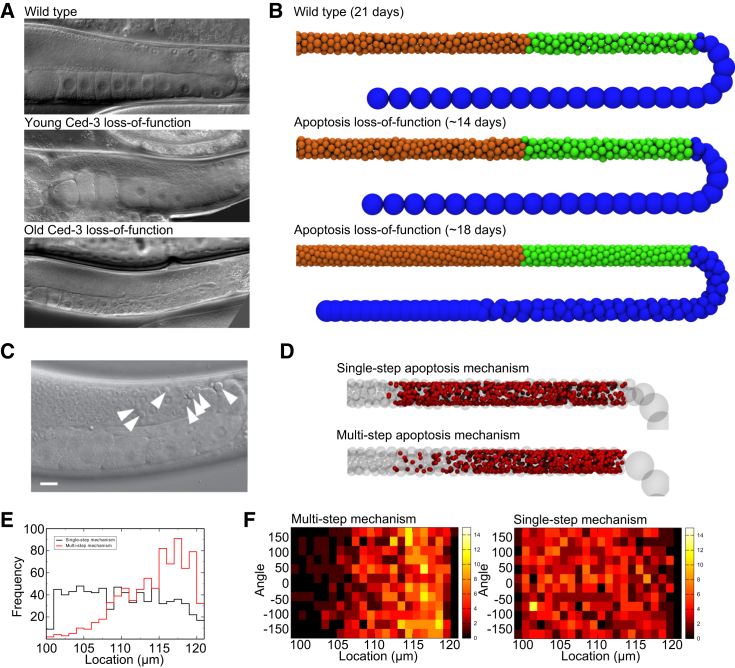
Effects of apoptosis mutations and alternative mechanisms. (*A*) Microscopy images showing germ cells in wild-type animals, and young and old Ced-3 loss-of-function mutations. (*B*) Wild-type models at 21 days show a distinct pattern of states across the length of the tube, with large oocytes in the distal arm and bend, after RAS downregulation. While apoptosis loss-of-function mutations appear normal at 14 days, the excess flow in cells caused by the loss from cell death causes smaller oocytes to move into the distal arm in older worms. Cells are colored by fate (*orange*, pachytene; *green*, diplotene; *blue*, diakinesis). (*C*) Microscopy image taken from Pinto and Hengartner (47) showing apoptosis preferentially occurring just before the bend (corpses indicated by *white arrows*). (*D*) Locations of apoptosis in two mechanisms of apoptosis (*red spheres*; *transparent spheres* show cell positions in a single frame, for reference). In a single-step model, cells randomly die and are removed from the simulation immediately, while in the multistep model cells randomly shrink until they reach a minimum size, and then die and are removed from the simulation. The single-step model results in deaths evenly distributed along the RAS activation zone, while in the multistep model cells die preferentially at the end of the RAS zone. (*E*) Histogram showing frequency of death as a function of location in the gonad. The RAS zone ranges from 100 *μ*m to the bend at 120 *μ*m. (*F*) Heat maps quantitatively showing the distribution of deaths around the tube for different death models. Deaths are evenly distributed across the radius of the tube.

Despite the importance of homeostasis in germline development, the precise purpose and mechanism of apoptosis in this system remains unclear. Loss-of-apoptosis mutations, such as *ced-3* loss-of-function alleles, lead to relatively mild phenotypes and no apparent overgrowth of germ cells (43). Young (≤14-day-old) loss-of-apoptosis mutants show normal morphology of the germline. In contrast, older apoptosis mutants, in which sperm supplies have been exhausted and oocytes cannot leave the proximal gonad arm, demonstrate an abnormal gonad morphology. While gonads of old wild-type animals still contain single-file, large stacked oocytes in the proximal arm, apoptosis mutants contain many smaller oocytes, tightly packed in multiple rows in the proximal arm (Fig. 6 *A*).

In our model, MAPK (represented by a Boolean value) directly activates a chain of proteins, which leads to apoptosis (also represented by a Boolean value). It has been observed that the relationship between MAPK and apoptosis is complex (19); MAPK is a hub for a number of signaling networks, and is known to be activated by other pathways (for example, in response to DNA damage (44,45)) and regulated by additional components (such as GLA-3 (18)). Moreover, it is not known if apoptosis is initiated after exit from the pachytene by an unknown signal or directly by RAS/MAPK signaling. Our executable model of cell signaling here does not aim to reproduce all the complex quantitative relationships between MAPK and its inputs, but rather the signal transduction process in the worm. However, modifying our model to make cell fate rather than MAPK activity drive apoptosis does not alter the observed behaviors. In the model, fate determination is driven by changes in gene expression, which is driven by RAS/MAPK activity. Therefore, a modification that causes MAPK-driven-fate changes simply adds more dependencies between MAPK and apoptosis, and does not change the relationship.

Analysis of the loss-of-apoptosis scenario highlighted the need for a negative feedback to limit mitotic growth. Early models of mitotic division allowed cells to divide without restriction. In the absence of fertilization (i.e., in female animals) and without apoptosis, cells would continue to divide despite the increasing overlap and forces between them, eventually leading to such a high pressure that cells would move through the gonad wall and rupture the gonadal tube. To prevent this, we included a negative feedback loop that stops mitotic growth if the pressure experienced by the cell exceeds a defined threshold.

Our model accurately reproduces the observed dynamic morphology of cell-death-defective (*ced*) mutants (Fig. 6, *A* and *B*). Once the germline is full of cells and fertilization has started, the germ cells can be seen to adopt a wild-type-like distribution of cell sizes, with the bend and proximal arm of the gonad filled with large, single-file oocytes. As mitotic divisions continue in the absence of apoptosis (while fertilization is ongoing), the forces generated are sufficient to force multiple germ cells into the bend. While repacking of the cells allows for some of them to reorder in young worms and grow to fill the tube, over time this leads to the influx of a larger number of small cells around the bend of the gonad and into the proximal arm. This ability of the cells to repack in three dimensions explains the distinct changes in morphology that occur as result of the flow-reducing effects of apoptosis. This observation may intuitively suggest that an increase in the rate of division (or a loss of fertilization) may lead to a morphology that resembles the loss-of-apoptosis phenotype, if the rate of division was able to overcome the rate of death. However, a limited exploration of models with different division rates suggests that the model is robust to changes in mitosis rates. This robustness arises because the packing of growing cells in the bend can act as a barrier to cell movement, forcing cells to remain in the growth-factor region for longer periods of time. This increases the likelihood of the cells dying, which in turn prevents an overflow of cells occurring as a result of mitosis.

The technical difficulties in experimentally observing and tracking the fates of individual germ cells over long time periods have made it difficult to study the exact causes of germ-cell death in the absence of external stress. It has been estimated that in the wild-type, roughly one-half of all germ cells die by apoptosis instead of differentiating into oocytes (15,16). Genetic studies have shown that increasing RAS/MAPK activity causes more germ-cell apoptosis, possibly due to an accelerated rate of pachytene exit (17,18,46). The mechanism by which individual cells are selected to survive or die is, however, not known. In our model, we have tested two possibilities of how cells may be selected for apoptosis. In the single-step model, all cells with active RAS have a defined probability of dying at any given timestep. Cell death immediately removes that cell from the simulation. (Fig. 6 *D*). The alternative multistep model defines a mechanism of cell death, where cells either shrink by a user-defined quantity and probability, dying when they reach a size threshold, or remain at the same size. Probabilities are assigned to each cell based on its rate of cell movement (i.e., time spent in the RAS region) to give at least a roughly 50% chance of cell death in a wild-type cell. The effect of loss-of-apoptosis mutations described above is insensitive to the precise mechanism of apoptosis.

Each of these two mechanisms has markedly different dynamics (Fig. 6, *D*–*F*). In both models, apoptosis reduces the flow rate and slows the development of oocytes relative to the loss-of-apoptosis mutation. In the single-step model for apoptosis, cell deaths are evenly distributed across the RAS activation zone (Fig. 6, *D*–*F*). In contrast, the multistep model for cell death leads to the majority of cells dying at the end of the RAS activation region. The location of cell death has been reported as at the start of the bend specifically (19,47). We therefore propose that cellular death consists of a multistep process, which increases the likelihood of entering apoptosis and leads to an accumulation of cell deaths at the end of the distal arm near the bend region. We further suggest that this multistep process may be achieved by a process of cell shrinking before apoptosis. For example, the cellularization and growth of germ cells entering oogenesis in the bend region may increase the local pressure and thus result the shrinking of adjacent cells, driving them into apoptosis. While this is only a single example of a multistep process, this would have the additional impact of selecting cells that enter pachytene for death based on their size when entering meiosis; smaller cells would require fewer steps to reach the apoptosis threshold and therefore would be more likely to die. As such, this could provide a mechanism for removing germ cells from the population that are underdeveloped. It should be noted, however, that there is, as of this writing, no experimental proof for such a mechanism.

#### Cellular flow in the gonad permits robust compartmentalization of cell fates

As the germ cells move along the gonad arms, they pass through four defined states based on their relative location in the different compartments: 1) mitosis in the distalmost region, which is activated by DELTA/NOTCH signaling; 2) entry into the pachytene stage of meiotic prophase I, once NOTCH signaling is terminated; 3a) entry into diplotene, which requires activation of the RAS/MAPK pathway by an unknown signal, followed by entry into diakinesis accompanied by oocyte formation in the turn region; 3b) a pro-apoptotic state as an alternative to entry into diakinesis; and 4) oocyte maturation at the proximal end of the gonad, which involves RAS/MAPK activation by a sperm signal. Therefore, the progression through these distinct fates must at least partially be defined by the changing environments the cells are exposed to in the different compartments of the gonad arms. We can observe this invariant fate progression in our model (Fig. 1 *B*). This is noteworthy as there are five fate variables in the model, which could potentially exist in 32 unique states. The correct progression observed in the model both demonstrates the model’s validity and raises the question of how the alternative potential fates are avoided to achieve invariance in fate progression across all wild-type animals.

**Figure 7:**
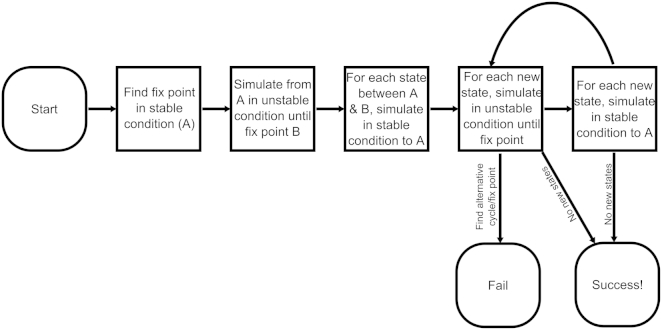
Proving all accessible states lead to a single fix point when moving from a stabilizing environment to an unstable environment. Cells at the border between two environments may move back and forth across the boundary due to diffusive motion. The different environments are represented by changes in constant values in the model (different conditions). All accessible states in the two different conditions are enumerated and tested to find whether they lead to the same fix point or not. This proceeds as follows: in the stable environment, the stable state is identified (shown as A). A simulation from state A in the unstable condition is performed until fix-point B is reached, and the set of states between A and B are collected. For each state, a simulation is performed in the stable condition until A is reached, and the set of states encountered in each simulation is recorded. If these have not been observed previously, simulations are performed in the unstable condition to determine if they reach fix-point B. This is repeated until either no new states are found (i.e., all accessible states have been identified), or an alternative fix point or cycle is discovered.

**Figure 8:**
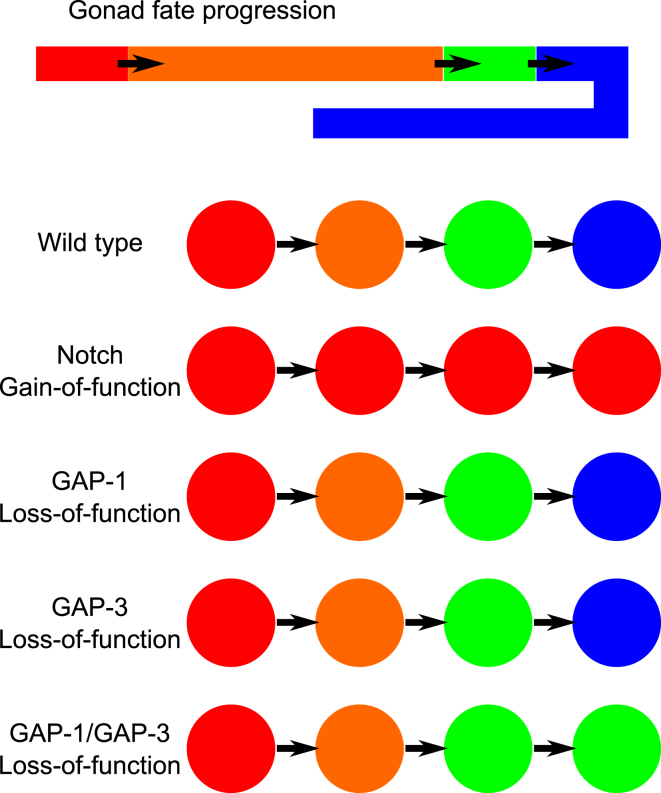
Fate progressions of germline cells. Germline cells follow an invariant developmental path from a mitotic state (*red*), to the pachytene stage I (*orange*), to diplotene (*green*), and finally to diakinesis (*blue*). This progression of fates observed in the hybrid model can be abstracted to an executable model, where we can characterize different mutations in terms of distinct fate progressions. This new executable model highlights that any instability of the germline cells in the absence of ligand can always lead to an invariant fate progression if the initial mitotic state is stable.

To address the question of how these compartments arise and what test conditions are known to disrupt this invariant pattern, we developed an executable model of the hybrid system. We represent each compartment in the cell as a distinct environment. For example, in the distal tip region, the external signal for DELTA is set to be active, while external ligands activating RAS are inactive. We then test the reachable states of the whole cell (including cell fates) for each compartment, based on the external signals and the reachable states in the previous compartment. In the first environment (the distal tip zone), we find that the model is stable—that is, all initial states eventually lead to a single final, mitotic state. From this state, cells move into a region without DELTA/NOTCH activation. The next environment lacks any external ligands, and analysis in the BMA demonstrates that in principle, when all states are considered initial, there are at least two possible end states, and an oscillation. However, we can prove that cells starting from the stable state in the DELTA/NOTCH active region lead to a single pachytene state, even if cells move repeatedly into and out of the DELTA zone (Fig. 7). This occurs because the stability of the model in the DELTA/NOTCH active state effectively reduces the accessible states when exiting the region. Through this mechanism, the stability of the initial environment can propagate to subsequent environments, and achieves an invariance in fate progression in the animal (Fig. 8).

This flow of cells through different compartments therefore allows complex decision-making processes between multiple end states to be encoded in both the protein network and the structure of the gonad. Our executable models also give us an opportunity to test alternative mechanisms of signaling in the gonad. While two external ligands are known (DELTA and major sperm protein (MSP)), the external ligand used to initiate RAS/MAPK activation has not been identified. Alternatively, an internal change within the cell may lead to RAS/MAPK activation, induced by timed events such as cell-cycle changes. Using our model, we studied how alternative mechanisms of signaling may achieve this.

Early models of the gonad included DELTA/NOTCH signaling, and an external signal that activates RAS/MAPK in the region before the bend. In this rudimentary model, maturation and fertilizations of germ cells occurred via a simplistic, disconnected pathway. While this model was capable of reproducing the invariant fate pattern across the gonad, individual cells at the first boundary between the early pachytene region and the RAS-active region transiently showed signs of later differentiation (i.e., late diakinesis). This occurred as a result of thermal motion in the system; cells could briefly move backward across the boundary and therefore initiate diakinesis.

This observation in the preliminary model is not supported by available experimental data. Furthermore, while it correctly describes the overall pattern of the fate progressions, it is incompatible with maturation of the cells being driven by a later activation of the MAPK. This is because models that have multiple, alternative fates caused by MAPK activation (entry into diakinesis versus maturation) would need to be bistable, and transient activations of late diakinesis may lead to early maturation of cells before the bend. This possibility is a property of the model, although we are not able to comment on the probability of the event due to intrinsic limitations of the model. Given that this outcome is possible but is never observed in nature, there must exist mechanisms to prevent this from arising. We propose three possible mechanisms by which this may be achieved: the first option is that fate determination at the boundary of the signaling regions must be highly buffered. That is to say, the signaling networks make RAS downregulation a slow process, in order to minimize the probability that backflow causes premature diakinesis. This buffering mechanism would need to be highly robust to the effects of reduced flow that increase the amount of time a cell resides at the boundary between signaling environments. The discovery of such a buffering mechanism would support this assertion. The second option is that a persistent MAPK activation is initiated by a timed event. And, finally, a third option is that a transient signal initiates a persistent MAPK activation. In the second and third options MAPK would need to be actively downregulated at the entry into diakinesis. Simulations show that if the exit from diakinesis is timed, small germ cells can escape into the proximal arm. One prediction arising from both the first and third possibilities would be that there was a clearly definable boundary where cells moved from one signaling region to another (similar to the boundary observed in the distal tip zone as cells exit mitosis). In contrast, if the initiation of MAPK activation is controlled by a timer (i.e., second option), cells may enter diplotene at slightly different locations in the same region of the gonad as the location of entry becomes dependent on the speed of an individual germ cell.

In light of this observation, and the role of MSP in activating RAS in oocyte maturation, both the hybrid model and QN were extended and further refined to explore both the timed activation of RAS in pachytene and MSP-induced activation. A new pathway was added to the QN to allow MSP to RAS via VAB1. To model the timed activation, the QN was modified to allow a transient input that causes RAS to become, and remain, active until it is downregulated by a later signal. In the hybrid model, this first transient input is initiated by the amount of time spent by in the pachytene, and the subsequent downregulation is caused by entry into the bend. These mechanisms exclude the possibility that any backflow could occur, and as such we propose that RAS is not activated by an external ligand. Such a timed event is tied to the meiotic cell cycle, which we suggest here as a mechanism of timing RAS activation. We further propose that subsequent RAS/MAPK downregulation mediated by GAP-1/GAP-3 and LIP-1 is linked to cellularization, as cells separate from the syncytium.

## Conclusions

The development of tools that model the interface between biological signaling networks and biophysical motion is an important challenge to understanding stem cells and organogenesis in a wide number of systems. Our approach takes two long-standing formalisms for modeling each of these phenomena, and has allowed us to connect them in a single model of oogenesis from stem cells in *C. elegans*. This allows us to gain insights into the intersection of cellular dynamics and signal transduction, which are inaccessible (as of this writing) by using experimental approaches, and has generated new predictions. Furthermore, we have also shown that a simple physical model, even based on limited data, in the hybrid model reproduces mutant behavior and predicts plausible physical parameters such as fertilization rates. Our tool and approach could therefore be easily applied and adapted to model a wide range of hybrid cellular phenomena. Finally, through the development of executable models of the hybrid model, we have shown a future route to allow the development of the organ to be integrated in yet larger systems. The use of a detailed executable model in combination with a physical model of the organ structure could be used to show how other organs and bodies within organisms interact and generate emergent properties. The future development and application of these hybrid models therefore offer unique opportunities for understanding complex development processes.

The predictions generated by this model demonstrate the type of unique information that can be offered by such hybrid approaches. The avoidance of clonal dominance by thermal mixing of the germ-cell population may represent an important mechanism for avoiding tumor development. While cancer is a widespread disease, the absolute likelihood of an individual cell of the trillions progressing to become cancerous is low, raising the question of why cancers are not more common given the number of opportunities to develop. The mixing of stem-cell populations would further minimize this by reducing the accumulation of mutations in populations. Our suggested multistep shrinkage mechanism for apoptosis gives two potentially new insights into the role and purpose of cell death in the gonad: it serves to reduce the flow rate, and it creates competition between stem cells, selectively killing smaller cells. This model is consistent with experimental evidence showing that increased RAS activity leads to smaller oocytes and increased cell death, in addition to other evidence presented here. Finally, the control of cell fate through cellular flow offers an explanation for a well-characterized phenomenon in the germline, allowing for future experimental examination. Together, these insights into stem-cell development in the germline demonstrate the power of our approach, and show how hybrid modeling may allow phenomena over multiple time- and length scales to be successfully combined.

## Author Contributions

B.A.H., N.P., A.H., and J.F. conceived and designed the experiments; B.A.H. performed the experiments; B.A.H., N.P., and J.F. analyzed the data; A.H. and B.A.H. contributed reagents/materials/analysis tools; and B.A.H., A.H., and J.F. wrote the article.

## Supporting Material

Document S1. One figure

Movie S1. Dynamic lineages One cell early in the simulation is selected, and its motions and the motions of its descendants are plotted as a line. Motions in the mitotic zone are random, while outside of the region cells move in a single direction along the length of the gonad. (*Blue sphere*) Births; (*red sphere*) deaths.

Movie S2. Dynamics of cell lineages over time All cells at a single time point are assigned a color, and from then onwards all descendants of these cells retain that color.

Document S2. Article plus Supporting Material

## References

[1] Kimble J., Crittenden S.L. (2005). Germline proliferation and its control. WormBook.

[2] Kimble J.E., White J.G. (1981). On the control of germ cell development in *Caenorhabditis elegans*. Dev. Biol..

[3] Lander A.D., Kimble J., Oskarsson T. (2012). What does the concept of the stem cell niche really mean today?. BMC Biol..

[4] Maciejowski J., Ugel N., Hubbard E.J. (2006). Quantitative analysis of germline mitosis in adult *C. elegans*. Dev. Biol..

[5] Crittenden S.L., Leonhard K.A., Kimble J. (2006). Cellular analyses of the mitotic region in the *Caenorhabditis elegans* adult germ line. Mol. Biol. Cell.

[6] Byrd D.T., Knobel K., Kimble J. (2014). A DTC niche plexus surrounds the germline stem cell pool in *Caenorhabditis elegans*. PLoS ONE.

[7] Crittenden S.L., Kimble J. (2008). Analysis of the *C. elegans* germline stem cell region. Methods Mol. Biol..

[8] Suh N., Crittenden S.L., Kimble J. (2009). FBF and its dual control of gld-1 expression in the *Caenorhabditis elegans* germline. Genetics.

[9] Kimble J. (2011). Molecular regulation of the mitosis/meiosis decision in multicellular organisms. Cold Spring Harb. Perspect. Biol..

[10] Lee M.H., Ohmachi M., Schedl T. (2007). Multiple functions and dynamic activation of MPK-1 extracellular signal-regulated kinase signaling in *Caenorhabditis elegans* germline development. Genetics.

[11] Hubbard E.J.A., Greenstein D. (2000). The *Caenorhabditis elegans* gonad: a test tube for cell and developmental biology. Dev. Dyn..

[12] Leacock S.W., Reinke V. (2006). Expression profiling of MAP kinase–mediated meiotic progression in *Caenorhabditis elegans*. PLoS Gen.

[13] Ohmachi M., Rocheleau C.E., Sundaram M.V. (2002). *C. elegans* ksr-1 and ksr-2 have both unique and redundant functions and are required for MPK-1 ERK phosphorylation. Curr. Biol.

[14] Cha D.S., Datla U.S., Lee M.H. (2012). The Ras-ERK MAPK regulatory network controls dedifferentiation in *Caenorhabditis elegans* germline. Biochim. Biophys. Acta.

[15] Galluzzi L., Joza N., Kroemer G. (2008). No death without life: vital functions of apoptotic effectors. Cell Death Differ..

[16] Bailly A., Gartner A. (2013). Germ cell apoptosis and DNA damage responses. Adv. Exp. Med. Biol..

[17] Rutkowski R., Dickinson R., Gartner A. (2011). Regulation of *Caenorhabditis elegans* p53/CEP-1-dependent germ cell apoptosis by Ras/MAPK signaling. PLoS Genet..

[18] Kritikou E.A., Milstein S., Hengartner M.O. (2006). *C. elegans* GLA-3 is a novel component of the MAP kinase MPK-1 signaling pathway required for germ cell survival. Genes Dev..

[19] Gartner A., Boag P.R., Blackwell T.K. (2008). Germline survival and apoptosis. WormBook.

[20] Killian D.J., Hubbard E.J. (2005). *Caenorhabditis elegans* germline patterning requires coordinated development of the somatic gonadal sheath and the germ line. Dev. Biol..

[21] Fisher J., Henzinger T.A. (2007). Executable cell biology. Nat. Biotechnol..

[22] Fisher J., Piterman N. (2010). The executable pathway to biological networks. Brief Funct. Genomics.

[23] Sadot A., Fisher J., Harel D. (2008). Toward verified biological models. *IEEE*/*ACM Trans*.

[24] Bonzanni N., Feenstra K.A., Krepska E. (2009). What can formal methods bring to systems biology?. FM 2009: Formal Methods.

[25] Kam N., Kugler H., Hubbard E.J.A. (2008). A scenario-based approach to modeling development: a prototype model of *C. elegans* vulval fate specification. Dev. Biol..

[26] Kam N., Cohen I.R., Harel D. (2001). The immune system as a reactive system: modeling T cell activation with statecharts. Human-Centric Computing Languages and Environments.

[27] Efroni S., Harel D., Cohen I.R. (2003). Toward rigorous comprehension of biological complexity: modeling, execution, and visualization of thymic T-cell maturation. Genome Res..

[28] Clark A., Galpin V., Hillston J. (2012). Formal methods for checking the consistency of biological models. Adv. Exp. Med. Biol..

[29] Clarke E.M., Grumberg O., Peled D.A. (1999). Model Checking.

[30] Beyer A., Eberhard R., Fisher J., Goryanin I.I., Goryachev A.B. (2012). A dynamic physical model of cell migration, differentiation and apoptosis in *Caenorhabditis elegans*. Advances in Systems Biology.

[31] Yamamoto T., Kanda N. (2012). Computational model for Brownian dynamics simulation of polymer/clay nanocomposites under flow. J. Non-Newt. Fluid Mech..

[32] Yamamoto T., Suga T., Mori N. (2005). Brownian dynamics simulation of orientational behavior, flow-induced structure, and rheological properties of a suspension of oblate spheroid particles under simple shear. Phys. Rev. E Stat. Nonlin. Soft Matter Phys..

[33] Rosser G., Baker R.E., Fletcher A.G. (2014). Modelling and analysis of bacterial tracks suggest an active reorientation mechanism in *Rhodobacter sphaeroides*. J. R. Soc. Interface.

[34] Ramis-Conde I., Drasdo D., Chaplain M.A.J. (2008). Modeling the influence of the E-cadherin-*β*-catenin pathway in cancer cell invasion: a multiscale approach. Biophys. J..

[35] Schaub M.A., Henzinger T.A., Fisher J. (2007). Qualitative networks: a symbolic approach to analyze biological signaling networks. BMC Syst. Biol..

[36] Gunsteren W., Berendsen H. (1982). Algorithms for Brownian dynamics. Mol. Phys..

[37] Benque D., Bourton S., Vardi M., Madhusudan P., Seshia S. (2012). BMA: visual tool for modeling and analyzing biological networks. Computer Aided Verification.

[38] Nawaz S., Sánchez P., Schaap I.A. (2012). Cell visco-elasticity measured with AFM and optical trapping at sub-micrometer deformations. PLoS ONE.

[39] van der Spoel, D., E. Lindahl, …, H. J. C. Berendsen. 2010. GROMACS User Manual, Ver. 4.6 Beta1. www.gromacs.org.

[40] Hess B., Kutzner C., Lindahl E. (2008). GROMACS 4: algorithms for highly efficient, load-balanced, and scalable molecular simulation. J. Chem. Theory Comput..

[41] Humphrey W., Dalke A., Schulten K. (1996). VMD: visual molecular dynamics. J. Mol. Graph..

[42] Fearon E.R., Vogelstein B. (1990). A genetic model for colorectal tumorigenesis. Cell.

[43] Gumienny T.L., Lambie E., Hengartner M.O. (1999). Genetic control of programmed cell death in the *Caenorhabditis elegans* hermaphrodite germline. Development.

[44] Wang Y., Wang S., Xie L. (2014). The roles of DNA damage-dependent signals and MAPK cascades in tributyltin-induced germline apoptosis in *Caenorhabditis elegans*. Chemosphere.

[45] Eberhard R., Stergiou L., Hengartner M.O. (2013). Ribosome synthesis and MAPK activity modulate ionizing radiation-induced germ cell apoptosis in *Caenorhabditis elegans*. PLoS Genet..

[46] Hajnal A., Berset T. (2002). The *C. elegans* MAPK phosphatase LIP-1 is required for the G(2)/M meiotic arrest of developing oocytes. EMBO J..

[47] Pinto S.M., Hengartner M.O. (2012). Cleaning up the mess: cell corpse clearance in *Caenorhabditis elegans*. Curr. Opin. Cell Biol..

