# Supplementary material for: Emergent Stem Cell Homeostasis in the C. elegans Germline Is Revealed by Hybrid Modeling
Source: Biophys J. 2015 Jul 21;109(2):428–38. doi: 10.1016/j.bpj.2015.06.007 (PMC4621618; doi:10.1016/j.bpj.2015.06.007)

Biophysical Journal

Supporting Material

Emergent Stem Cell Homeostasis in the *C. elegans* Germline Is Revealed by Hybrid Modeling

Benjamin A. Hall,[1,2,]* Nir Piterman,[3] Alex Hajnal,[4] and Jasmin Fisher[2,5,]*

[1]Medical Research Council Cancer Unit, Hutchison/Medical Research Council Research Centre, University of Cambridge, Cambridge, United Kingdom; [2]Microsoft Research Cambridge, Cambridge, UK; [3]Department of Computer Science, University of Leicester, Leicester, UK; [4]Institute of Molecular Life Sciences, University of Zurich, Zurich, Switzerland; and [5]Department of Biochemistry, University of Cambridge, Cambridge, UK

Supplementary information

Supplementary Figure 1.

Birth, death and fertilisation over time. Over the 3 week lifetime of the animal 150 oocytes mature per arm of the gonad, with roughly one cell maturing per ~23 minutes. Here we observe exhaustion of the sperm after 4 weeks, and one cell maturing every ~60 minutes.

Supplementary Video 1.

Dynamic lineages. One cell early in the simulation is selected, and its motions and the motions of its descendants are plotted as a line. Motions in the mitotic zone are random, whilst outside of the region cells move in a single direction along the length of the gonad. Births are indicated by a blue sphere in the path, whilst deaths are indicated by a red sphere.

Supplementary Video 2.

Dynamics of cell lineages over time. All cells at a single time point are assigned a colour, and from then onwards all descendants of these cells retain the same colour.

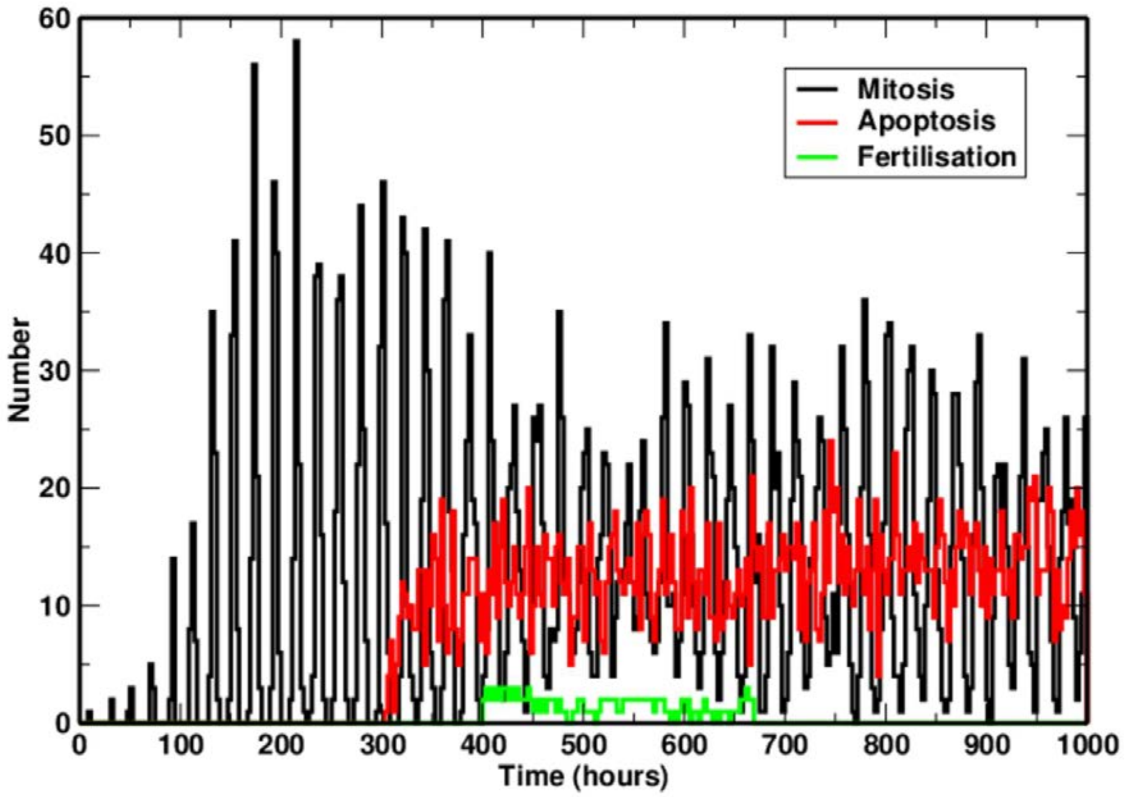

Supplement: Document S1. One figure [file mmc1.pdf]
