# Supplementary figures and images for: Emergent Stem Cell Homeostasis in the C. elegans Germline Is Revealed by Hybrid Modeling
Source: Biophys J. 2015 Jul 21;109(2):428–38. doi: 10.1016/j.bpj.2015.06.007 (PMC4621618; doi:10.1016/j.bpj.2015.06.007)

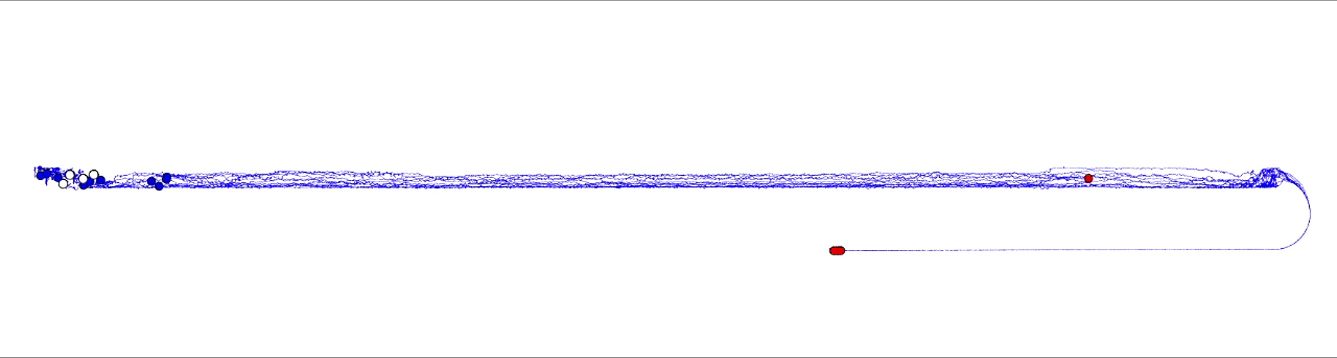

Supplement: Movie S1. Dynamic lineages — One cell early in the simulation is selected, and its motions and the motions of its descendants are plotted as a line. Motions in the mitotic zone are random, while outside of the region cells move in a single direction along the length of the gonad. (Blue sphere) Births; (red sphere) deaths. [file mmc2.jpg]

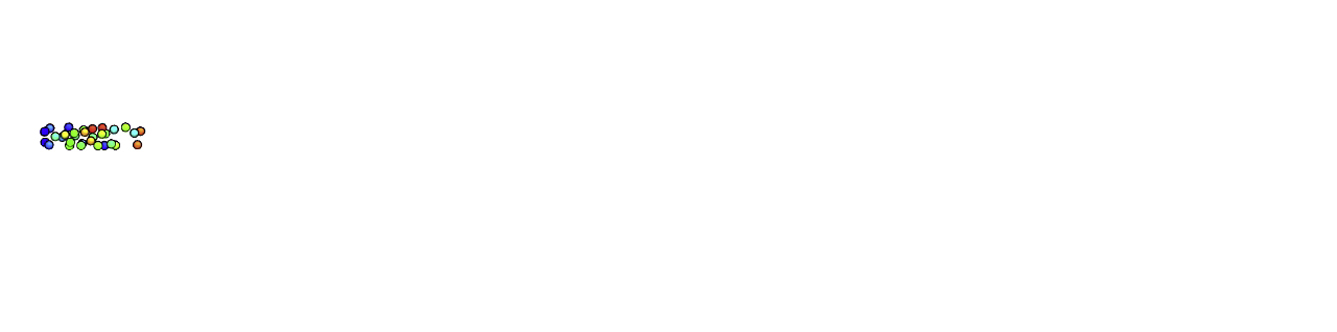

Supplement: Movie S2. Dynamics of cell lineages over time — All cells at a single time point are assigned a color, and from then onwards all descendants of these cells retain that color. [file mmc3.jpg]
